# SARS-CoV-2 Membrane Protein Inhibits Type I Interferon Production Through Ubiquitin-Mediated Degradation of TBK1

**DOI:** 10.3389/fimmu.2021.662989

**Published:** 2021-05-18

**Authors:** Liyan Sui, Yinghua Zhao, Wenfang Wang, Ping Wu, Zedong Wang, Yang Yu, Zhijun Hou, Guangyun Tan, Quan Liu

**Affiliations:** ^1^ Laboratory of Emerging Infectious Disease, Institute of Translational Medicine, The First Hospital of Jilin University, Changchun, China; ^2^ College of Basic Medical Science, Jilin University, Changchun, China; ^3^ College of Wildlife and Protected Area, Northeast Forestry University, Harbin, China; ^4^ Hospital of Stomatology, Jilin University, Changchun, China; ^5^ Department of Immunology, Institute of Translational Medicine, The First Hospital of Jilin University, Changchun, China; ^6^ School of Life Sciences and Engineering, Foshan University, Foshan, China

**Keywords:** SARS-CoV-2, membrane protein, type I interferon, TBK1, ubiquitination

## Abstract

The severe acute respiratory syndrome coronavirus-2 (SARS-CoV-2) is the causative pathogen of current COVID-19 pandemic, and insufficient production of type I interferon (IFN-I) is associated with the severe forms of the disease. Membrane (M) protein of SARS-CoV-2 has been reported to suppress host IFN-I production, but the underlying mechanism is not completely understood. In this study, SARS-CoV-2 M protein was confirmed to suppress the expression of IFNβ and interferon-stimulated genes induced by RIG-I, MDA5, IKKϵ, and TBK1, and to inhibit IRF3 phosphorylation and dimerization caused by TBK1. SARS-CoV-2 M could interact with MDA5, TRAF3, IKKϵ, and TBK1, and induce TBK1 degradation *via* K48-linked ubiquitination. The reduced TBK1 further impaired the formation of TRAF3–TANK–TBK1-IKKε complex that leads to inhibition of IFN-I production. Our study revealed a novel mechanism of SARS-CoV-2 M for negative regulation of IFN-I production, which would provide deeper insight into the innate immunosuppression and pathogenicity of SARS-CoV-2.

## Introduction

The newly emerged severe acute respiratory syndrome coronavirus 2 (SARS-CoV-2) that causes the coronavirus disease 2019 (COVID-19) pandemic is a novel viral member in the genus *Betacoronavirus* of the family *Coronaviridae* (1, 2), which is the third coronavirus associated with severe respiratory diseases, following SARS-CoV and Middle East respiratory syndrome coronavirus (MERS-CoV) (3, 4). As of January 25, 2021, there are more than 100 million confirmed cases of COVID-19, with 2 million deaths all over the world (https://coronavirus.jhu.edu/). SARS-CoV-2 has a single stranded, positive-sense RNA genome, which contains approximately 29.7 kb nucleotides, with at least 12 open reading frames (ORFs) encoding 16 nonstructural proteins (NSPs), seven accessory proteins and four structural proteins (envelope, spike, membrane, and nucleocapsid) (1, 5).

Innate immune response is considered as the first host defense against viral infections, which initiates antiviral responses through the pattern recognition receptors (PRRs) of hosts. The double-strand RNA, resulting from coronavirus genome replication and transcription, is first recognized by host PRRs, including the retinoic acid-inducible gene-I (RIG-I) like receptors (RLRs), such as RIG-I and melanoma differentiation associated gene 5 (MDA5) (6, 7). Activated RLRs trigger TANK-binding kinase 1 (TBK1) activation through the key adaptor mitochondrial antiviral signaling (MAVS) (8), further activating the transcription factor interferon regulation factor 3 (IRF3) to induce production of type I interferon (IFN-I) and downstream interferon-stimulated genes (ISGs), the critical host antiviral factors (9, 10).

Viruses have evolved elaborate mechanisms to evade host antiviral immunity, with a common strategy of virus-encoded IFN antagonists (11). SARS-CoV-2 encoded proteins, such as ORF6, NSP13, membrane (M), and nucleocapsid (N) proteins have been shown to possess the IFN-antagonizing properties (12–14). The SARS-CoV-2 M protein can interact with MAVS and impede the formation of MAVS–TRAF3–TBK1 complex to antagonize IFN-I production (15, 16). However, whether SARS-CoV-2 M interacts with RIG-I, MDA5, or TBK1 is in dispute (15, 16), and its association with TRAF3 and IKKϵ remains to be investigated, which would contribute to understanding of the immune evasion mediated by the SARS-CoV-2 M protein.

In this study, we reported that the SARS-CoV-2 M protein suppressed IFN-I production by interacting with TBK1 and promoting its degradation *via* K48-linked ubiquitination, and M protein could also interact with MDA5, TRAF3 and IKKϵ. The reduced TBK1 impaired the formation of TRAF3–TANK–TBK1-IKKε complex, resulting to the inhibition of IRF3 activation and further IFN-I production. This study reveals a novel mechanism for SARS-CoV-2 M protein to inhibit IFN-I production, which provides in-depth insight into the innate immunosuppression and pathogenicity of SARS-CoV-2.

## Materials and Methods

### Plasmids

The SARS-CoV M protein (NC_004718), SARS-CoV-2 M protein of IPBCAMS-WH-01/2019 strain (no. EPI_ISL_402123), TBK1 genes and their truncations were cloned into vector VR1012 with the Flag-tag or GST-tag (Sangon Biotech, Shanghai, China). The expression vectors of Flag-Ubi, Flag-K48-Ubi, Flag-K63-Ubi, pIFNβ-Luc, ISRE-luc and Renilla luc were constructed in the previous study (17, 18). The expression plasmids for IRF3, TANK, IKKϵ, RIG-I and TRAF3 were purchased from PPL Biotech, Jiangsu, China. The expression plasmid for TBK1 and MDA5 was purchased from Miaoling Biotech, Wuhan, China.

### Antibodies and Drugs

Anti-Flag, anti-HA, anti-Myc, anti-GST tag antibodies, anti-Phospho-IRF3 (S396) antibody, anti-GAPDH and anti-actin antibodies, CoraLite594-conjugated goat anti‐rabbit IgG, and CoraLite488-conjugated Goat Anti-Rabbit IgG antibodies were purchased from Proteintech, Wuhan, China; anti-IRF3 antibody was obtained from the Cell Signaling, Danvers, USA. MG132 was purchased from Sigma, St Louis, USA. Z-VAD-FMK was obtained from Promega, Madison, USA. Chloroquine was purchased from MCE, Monmouth, USA.

### ELISA

Flag tagged empty vector or Flag-M and MDA5, TBK1 or IKKϵ expression plasmids were co-transfected into HEK293T cells. After 24 h, culture supernatant was harvested and secreted IFNβ was detected by ELISA according to the manufacturers’ protocol from Proteintech, Wuhan, China.

### Luciferase Reporter Assay

HEK293T cells (2 × 10^5^, 24-well plate) were transfected with reporter plasmid of 200 ng IFNβ-Luc or ISRE-Luc and 40 ng Renilla-Luc, together with expressing plasmids of RIG-I, MDA5, TBK1, IKKϵ, or 1 μg/ml poly(I:C), and plasmids expressing viral proteins. Cells were harvested after 24 h, and cell lysates were used to determine individually for IFNβ or ISRE luciferase using a Dual Luciferase Reporter Assay System (Promega, Madison, USA). Relative luciferase activity was calculated by normalize firefly luciferase activity to Renilla luciferase activity recovered from cell lysate.

### RNA Isolation and Quantitative PCR (qPCR)

The cDNA was synthesized after total RNA extraction. Quantitative cDNA amplification was performed using ABI Plus one (Applied Biosystems, Foster City, USA); primers used were listed in **Supplementary Table 1**. Each PCR reaction of 20 µl contains 8 µl of ddH_2_O, 10 µl of SYBR green premix, 1 µl of cDNA, and 0.5 µl of each forward and reverse primer (10 µM). Amplification conditions were as follows: 5 min denaturation at 95°C, 40 cycles of PCR for the quantitative analysis (95°C for 10 s and 60°C for 30 s). The relative expression of each gene was analyzed by 2^−ΔΔCT^ method.

### Immunofluorescence

To assess the colocation of SARS-CoV-2 M with TRAF3, TBK1 and IKKϵ, an immunofluorescence assay was carried out as described elsewhere (19). After fixed with 4% paraformaldehyde for 30 min, the cells were permeabilized with 1% Triton X-100/PBS for 15 min and blocked with blocking buffer (PBS +1% bovine serum albumin) for 1 h, cells were then incubated with primary and secondary antibodies and stained for nuclear. Fluorescence was captured on OLYMPUS FV3000 confocal microscope (Olympus, Shinjuku, Japan).

### Co-Immunoprecipitation and Immunoblot Analysis

Co-immunoprecipitation and immunoblot analysis were performed as described elsewhere (18). Briefly, cells were lysed with cell lysis buffer and boiled for 10 min. The cell lysates added anti-Flag or anti-HA agarose were incubated on a roller at 4°C overnight. The immunoprecipitants or cell lysates were subjected to electrophoresis on a 12% SDS-PAGE gel and transferred onto PVDF membranes for immunoblot analysis. The membranes were blocked and incubated with primary and HRP-conjugated secondary antibodies. Chemi-luminescence was tested using ECL (Thermo, Waltham, USA) and protein bands were visualized by Biorad CHemiDoc XRS (Biorad, California, USA).

### Statistical Analysis

Data were analyzed by one-way analysis of variance (ANOVA) with Dunnett’s correction using the GraphPad Prism5 statistical software (Graphpad Software, San Diego, USA). All data were expressed as mean ± SE. P value less than 0.05 was considered statistically significant, and less than 0.01 and 0.001 was considered extremely significant.

## Results

### SARS-CoV-2 M Protein Inhibits RIG-I/MDA5/IKKϵ/TBK1-Mediated IFN-I Signaling

We first tested the influence of SARS-CoV-2 M protein on the induction of IFNβ and downstream ISGs expression (**Figure S1**). The luciferase reporter assay showed that the SARS-CoV-2 M protein significantly inhibited IFNβ and ISRE promoter activities induced by poly(I:C) (**Figures S1B, C**), and mRNA expression of IFN-I (*IFNα*, *IFNβ*) and ISGs (*ISG15*, *OAS1* and *SOCS1*) genes was also repressed by the SARS-CoV-2 M protein (**Figure S1D**). These results indicated that SARS-CoV-2 M can inhibit IFN-I production, which are consistent with the previous report (15).

**Figure 1:**
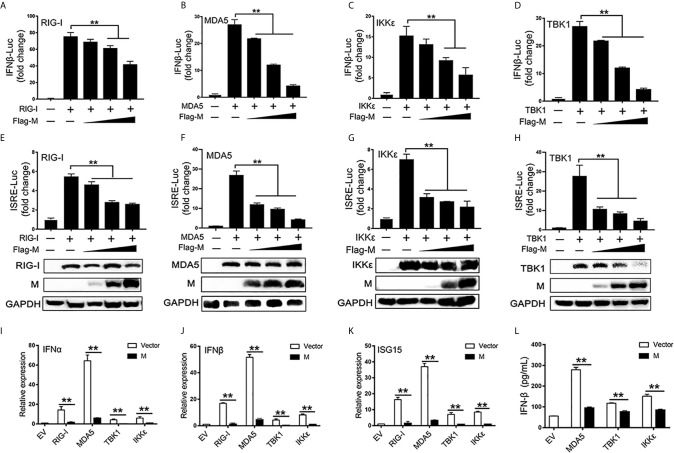
The SARS-CoV-2 M inhibits IFN-I signaling. **(A–H)** HEK293T cells in 24-well plate were transfected with an IFNβ or ISRE reporter plasmid, along with a control plasmid or with increasing amount of Flag-M (0.2, 0.4, and 0.8 μg), together with plasmids expressing RIG-I **(A, E)**, MDA5 **(B, F)**, IKKϵ **(C, G)**, or TBK1 **(D, H)**. At 24 h post-transfection, luciferase activity was measured. The expression levels of indicated proteins were analyzed by immunoblot. **(I–K)**. Flag tagged empty vector or Flag-M and RIG-I, MDA5, TBK1 or IKKϵ expression plasmids were co-transfected into HEK293T cells. After 20 h, cells were harvested and relative mRNA levels of type I IFN and ISGs genes expression were analyzed by qPCR. GAPDH was used as a normalizer. **(L)**. Flag tagged empty vector or Flag-M and MDA5, TBK1 or IKKϵ expression plasmids were co-transfected into HEK293T cells. After 24 h, culture supernatant was harvested and secreted IFNβ was detected by ELISA. Bars represent the mean of three biological replicates (n = 3) and all data are expressed as mean ± SE. **p < 0.01.

RIG-I and MDA5 are key sensors of RNA virus infection, which play critical roles in coronavirus recognition and IFN-I signaling activation (20). We then explored the effect of SARS-CoV-2 M on RIG-I signaling. RIG-I/MDA5/IKKϵ and increasing amounts of M expression plasmids were co-transfected to HEK293T cells, the activation of the IFNβ and ISRE promoter were tested by luciferase reporter assay. Co-expression of SARS-CoV-2 M suppressed both IFNβ and ISRE promoter activation induced by RIG-I, MDA5, and IKKϵ in a dose dependent manner (**Figures 1A–C, E–G**). We further demonstrated that the IFNβ and ISRE promoter activity induced by TBK1 were significantly decreased in SARS-CoV-2 M transfected cells (**Figures 1D, H** **)**. Additionally, SARS-CoV-2 M suppressed ISRE promoter activity to a greater extent than that of IFNβ induced by RIG-I signaling. This may be explained by that M can block the phosphorylation of STAT1, a key step for ISGs production in the downstream of interferon signaling (13). We further detected the effect of M protein on RIG-I/MDA5/IKKϵ/TBK1 induced IFN-I and ISGs mRNA expression and secreted IFNβ expression. QPCR results showed that M protein significantly inhibited IFNα, IFNβ and ISG15 mRNA expression induced by RIG-I, MDA5, TBK1 and IKKϵ (**Figures 1I–K**). ELISA results showed that IFNβ induced by MDA5, TBK1 and IKKϵ was significantly inhibited by M protein (**Figure 1L**).These results indicate that SARS-CoV-2 M inhibits RIG-I/MDA5/IKKϵ/TBK1-mediated IFN-β and ISGs production.

### SARS-CoV-2 M Protein Antagonizes IRF3 Activation

**Figure 2:**
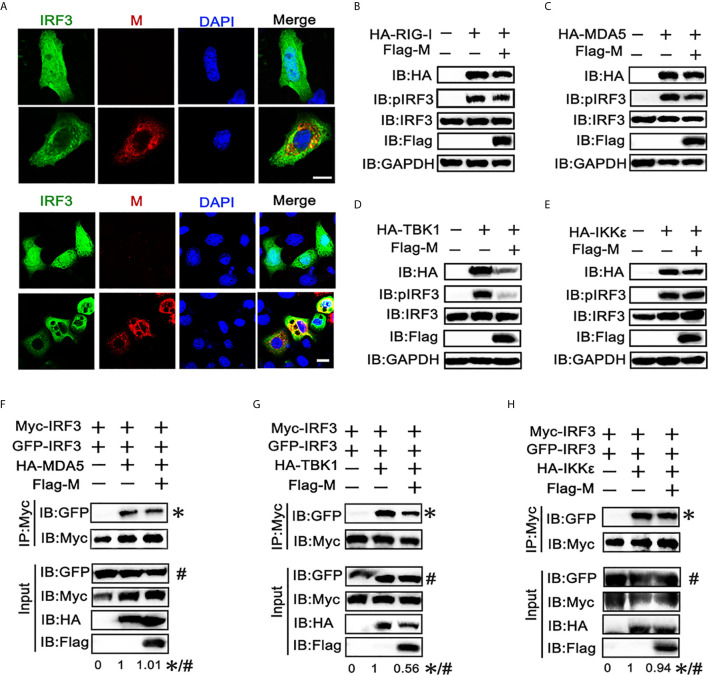
SARS-CoV-2 M inhibits IRF3 nuclear translocation, phosphorylation and dimerization. **(A)** HEK293T cells were co-transfected with EGFP-tagged IRF3 and a control plasmid or Flag-M expression plasmid. After 24 h, cells were transfected with poly(I:C) for 6 h, and were immuno‐stained with anti‐Flag antibody (red) and counterstained with DAPI to examine chromosomes (blue). Green: IRF3 signal; Red: SARS-2-CoV-2 M signal; Blue: DAPI (nuclei signal). **(B–E)** HEK293T cells were transfected with Flag-M together with HA-tagged RIG-I **(B)**, MDA5 **(C)**, TBK1 **(D)**, or IKKϵ **(E)**, which was applied to activate IRF3. Proteins were extracted 24 h after transfection. The indicated proteins were analyzed by immunoblot. GAPDH was detected as a loading control. **(F–H)** HEK293T cells were transfected with GFP-IRF3, Myc-IRF3, and Flag-M together with HA-tagged MDA5 **(F)**, TBK1 **(G)**, or IKKϵ **(H)**, which was applied to activate IRF3 dimerization. Proteins were extracted 30 h after transfection. The cell lysates were immunoprecipitated with anti-Myc antibody. The cell lysates and the immunoprecipitants were analyzed by immunoblot. The relative band intensity (*/#) of co-immunoprecipitated IRF3 in **(F–H)** was measured using ImageJ software.

The phosphorylation and nuclear translocation of IRF3 is the key step for IRF3 activation and IFN production (21). Thus, we explored the effect of M protein on IRF3 activation. Immunofluorescence showed that poly(I:C) triggered nuclear translocation of IRF3 was impeded in cells overexpressing SARS-CoV-2 M (**Figure 2A**). Next, we examined whether M protein affects RIG-I, MDA5, TBK1 and IKKϵ induced phosphorylation of IRF3. HEK293T cells were co-transfected with HA-RIG-I, HA-MDA5, HA-TBK1, or HA-IKKϵ in the presence or absence of Flag-M. We found that stimulation of HEK293T cells with RIG-I, MDA5, or TBK1 alone triggered the phosphorylation of IRF3 (**Figures 2A–C**). Co-expression of M slightly reduced the phosphorylation of IRF3 which was activated by RIG-I and MDA5 (**Figures 2B, C** **)**, while the phosphorylated IRF3 was almost undetectable in TBK1-induced group (**Figure 2D**). Overexpression of IKKϵ induced the phosphorylation of IRF3, but SARS-CoV-2 M did not change the amount of phosphorylated IRF3 induced by IKKϵ (**Figure 2E**). We then detected IRF3 dimerization by co-immunoprecipitation. Results showed that MDA5, TBK1 and IKKϵ could elevate the dimerization of IRF3, and M protein significantly inhibited the dimerization of IRF3 triggered by TBK1 (**Figure 2G**). However, M protein showed no significant impact on the IRF3 dimerization triggered by MDA5 or IKKϵ (**Figures 2F, H** **)**.These results suggested that SARS-CoV-2 M protein can prevent IRF3 nuclear translocation and inhibit IRF3 phosphorylation and dimerization induced by TBK1.

We also found that the expression level of TBK1 was significantly decreased when co-expressed with SARS-CoV-2 M, while M co-transfection did not significant affect the expression of other RLR signaling molecules. The same phenomenon was also observed in the immunoblot results (**Figures 1E–G**), indicating that the TBK1 expression may be inhibited by the SARS-CoV-2 M protein.

### SARS-CoV-2 M Inhibits IFN-I Production by Promoting TBK1 Degradation

**Figure 3:**
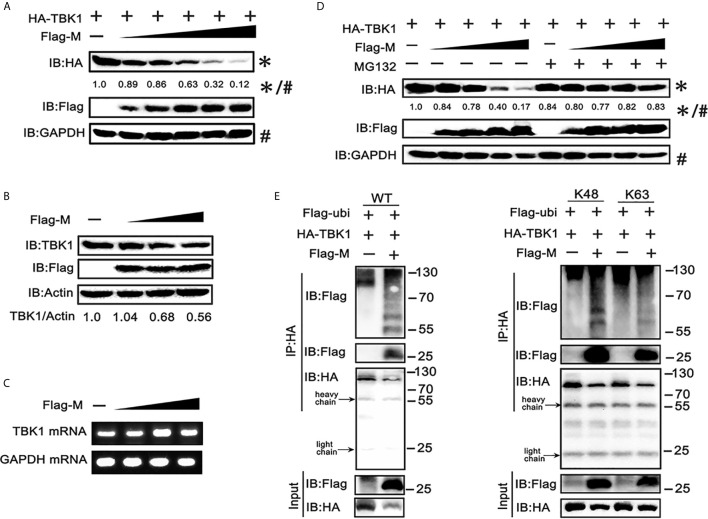
SRAS-CoV-2 M degrades TBK1 *via* ubiquitination. **(A)** HEK293T cells in 6-well plate were transfected with a control plasmid or increasing doses of plasmid expressing Flag-M (0.25, 0.5, 1, 2 and 4 μg), along with HA-TBK1 plasmids for 24 h. The cell lysates were analyzed by immunoblot. GAPDH was detected as a loading control. **(B)** HEK293T cells in 24-well plate were transfected with a control plasmid or increasing doses of Flag-M (0.25, 0.5, 1 μg) for 24 h. The cell lysates were analyzed by immunoblot. Actin was detected as a loading control. **(C)** HEK293T cells in 24-well plate were transfected with a control plasmid or increasing dose of Flag-M (0.25, 0.5, 1 μg). After 24 h, cells were harvested and mRNA levels of TBK1 genes expression in empty vector or M plasmids transfection groups were analyzed by qPCR. GAPDH was used as a normalizer. **(D)** HEK293T cells were transfected with a control plasmid or increasing doses of Flag-M and HA-TBK1 plasmids. After 24 h, cells were treated with 20 μM MG132 or DMSO for 6 h. The cell lysates were analyzed by immunoblot. **(E)** Co-immunoprecipitation and immunoblot analysis of extracts from HEK293T cells transfected with TBK1 with or without SARS-CoV-2 M as well as Flag-Ub (WT, K48-linked, or K63-linked) expression plasmids as indicated. The relative band intensity (*/#) of HA-TBK1 in **(A, D)** was measured using ImageJ software.

To further explore the mechanism responsible for the decreased expression of TBK1 in SARS-CoV-2 M co-expressed cells, we co-transfected TBK1 and a dose gradient of SARS-CoV-2 M into HEK293T cells. The immunoblot results showed that the expression of exogenous TBK1 was gradually decreased accompanied by the increased amount of SARS-CoV-2 M (**Figure 3A**). The M protein also degraded the endogenous TBK1, with a lower efficiency compared to the overexpressed TBK1 (**Figure 3B**), probably due to the low level of endogenous TBK1. In contrast, TBK1 mRNA level was not changed upon overexpression of SARS-CoV-2 M (**Figure 3C**), indicating that TBK1 may be degraded at the protein level.

Ubiquitination, a multifunctional post-translational modification, plays critical roles in the regulation of antiviral innate immune responses (22). The lysine 63 (K63)-linked ubiquitination of TBK1 facilitates its activation, whereas K48-linked ubiquitination mediates its proteasomal degradation and terminates the downstream signaling (23). To investigate if TBK1 is degraded by SARS-CoV-2 M *via* ubiquitination modification, we added proteasome inhibitor MG-132, and results showed that SARS-CoV-2 M-induced TBK1 degradation was blocked (**Figure 3D**), while caspase inhibitor (Z-VAD-FMK) and lysosome inhibitor (chloroquine) showed no significant impact on degradation of TBK1 induced by M protein (**Figure S2**), suggesting that TBK1 was targeted for proteasomal degradation by SARS-CoV-2 M. We further characterized SARS-CoV-2 M-mediated ubiquitination of TBK1, and found that SARS-CoV-2 M induced an increased K48-linked ubiquitination of TBK1, whereas K63-linked ubiquitination remained unchanged (**Figure 3E**). An interaction between TBK1 and SARS-CoV-2 M was also detected in the ubiquitin co-immunoprecipitation test (**Figure 3E**). Taken together, these results suggested that SARS-CoV-2 M promoted TBK1 degradation *via* K48-linked ubiquitination.

### SARS-CoV-2 M Protein Interacts With MDA5/TRAF3/TBK1/IKKϵ

**Figure 4:**
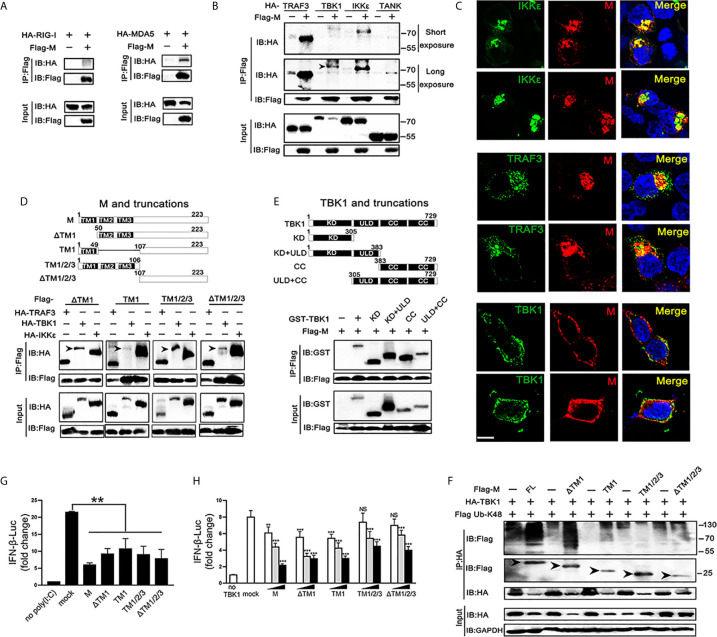
Association and colocalization of SRAS-CoV-2 M with MDA5, TRAF3, TBK1 and IKKϵ. **(A, B)** HEK293T cells were transfected with a control plasmid or Flag-M, along with HA-tagged plasmids expressing RIG-I, MDA5, TRAF3, TBK1, IKKϵ and TANK as indicated for 30 h. The cell lysates were immunoprecipitated with anti-Flag antibody. The cell lysates and the immunoprecipitants were analyzed by immunoblot. **(C)** HEK293T cells were co-transfected with a control plasmid or Flag-M and HA-tagged TRAF3, TBK1 and IKKϵ expression plasmids for 24 h. The cells were fixed and subjected to immunofluorescence analysis to detect M (red) and TRAF3, TBK1 and IKKϵ (green) using anti-Flag and anti-HA antibodies, respectively. The cell nuclei were stained with DAPI (blue). Co-localization appeared yellow. Green: TRAF3/TBK1/IKKϵ signal; Red: SARS-2-CoV-2 M signal; Blue: DAPI (nuclei signal). *Bar*, 20 μm. **(D)** Co-immunoprecipitation and immunoblot analyses of the indicated proteins in HEK293T cells transfected with various SARS-CoV-2 M truncated fragments along with TRAF3, TBK1 and IKKϵ. TM, trans-membrane domain. Numbers above the domain names indicate amino acid positions. **(E)** Co-immunoprecipitation and immunoblot analyses of the indicated proteins in HEK293T cells transfected with various TBK1 truncated fragments and SARS-CoV-2 M as indicated. KD, kinase domain; ULD, ubiquitin-like domain; CC, coiled-coil domain. **(F)** Flag-M and its truncations expression plasmids were co-transfected with an IFN-β reporter plasmid, along with a control plasmid and 1 μg/ml poly(I:C). After 24 h, cells were harvested for luciferase reporter assay. **(G)** Flag-M and its truncations expression plasmids were co-transfected with an IFN-β reporter plasmid, along with TBK1 expression plasmid. After 24 h, cells were harvested for luciferase reporter assay. **(H)** Co-immunoprecipitation and immunoblot analysis were conducted on the extracts from HEK293T cells transfected with TBK1 with or without full length (FL) or truncated SARS-CoV-2 M as well as K48-linked ubiquitin as indicated. Arrows indicated the full length or truncated M proteins detected in immunoprecipitants. Bars represent the mean of three biological replicates (n=3) and all data are expressed as mean ± SE. NS: nonsense. **p < 0.01, ***p < 0.001.

TBK1 can form a polyprotein complex with TRAF3, TANK and IKKϵ in the cytoplasm, which is a crucial step in the IRF3 activation (24, 25). As SARS-CoV-2 M represses the production of IFNβ induced by RIG-I, MDA5, TBK1 and IKKϵ, we further explored if the SARS-CoV-2 M protein associates with RIG-I, MDA5, and TRAF3–TANK–TBK1/IKKϵ complex. We overexpressed SARS-CoV-2 M and the transducer protein RIG-I, MDA5, TRAF3, TBK1, IKK*ϵ*, or TANK in HEK293T cells and co-immunoprecipitation was performed to detect their interactions (**Figures 4A, B** **)**. Although both RIG-I and MDA5 were expressed abundantly in HEK293T cells, only MDA5 was found to co-precipitate with SARS-CoV-2 M (**Figure 3A**). For the TRAF3–TANK–TBK1/IKKϵ complex, the results showed that SARS-CoV-2 M was specifically co-precipitated with TRAF3, TBK1 and IKK*ϵ*, but not TANK, and immunoblot detection of TBK1 can only be tested for long exposure time because of the severely reduced TBK1 (**Figure 4B**). Immunofluorescence analyses showed that SARS-CoV-2 M co-localized substantially with TRAF3, IKK*ϵ*, and TBK1 in HEK293T cells to discrete cytoplasmic subdomains (**Figure 4C**). These results indicated that SARS-CoV-2 M interacts with the MDA5, TRAF3, TBK1 and IKK*ϵ*.

We further addressed which domains of SARS-CoV-2 M are required for its interaction with TRAF3, TBK1, and IKK*ϵ*. We constructed plasmids expressing various truncated fragments of SARS-CoV-2 M. The association of SARS-CoV-2 M mutants with TRAF3, TBK1 and IKK*ϵ* was assessed by co-transfection of TRAF3, TBK1 and IKK*ϵ* and each of the SARS-CoV-2 M mutants into HEK293T cells. All the examined SARS-CoV-2 M truncations interacted with TRAF3, TBK1, and IKKϵ (**Figure 4D**), suggesting that the SARS-CoV-2 M could interact with TRAF3, TBK1, and IKKϵ *via* its different fragments. Consistently, luciferase reporter assay showed that the all the truncations of the SARS-CoV-2 M were able to inhibit the promoter activation of IFNβ induced by poly(I:C) (**Figure 4F**). The coiled-coil domain of TBK1 was shown as the strongest that interacts with SARS-CoV-2 M, followed by the kinase domain (**Figure 4E**).

We then investigated the effect of SARS-CoV-2 M truncations on TBK1-induced IFNβ activity and TBK1 expression. Luciferase reporter assay showed that all the SARS-CoV-2 M truncations could inhibit TBK1-induced INFβ activity (**Figure 4G**), while only ΔTM1 and TM1 truncations induced TBK1 degradation (**Figure S3**). Consistently, co-immunoprecipitation results showed that only the ΔTM1 and TM1 truncations induced K48-linked ubiquitination of TBK1, while the TM1/2/3 or ΔTM1/2/3 showed no impact on TBK1 ubiquitination level (**Figure 4H**). A possible explanation is that the ubiquitination of TBK1 by SARS-CoV-2 M may need a cooperation of multiple domains, while only N or C terminal of SARS-CoV-2 M cannot elevate ubiquitination level of TBK1.

### SARS-CoV-2 M Impairs the TRAF3–TANK–TBK1–IKKϵ Complex

**Figure 5:**
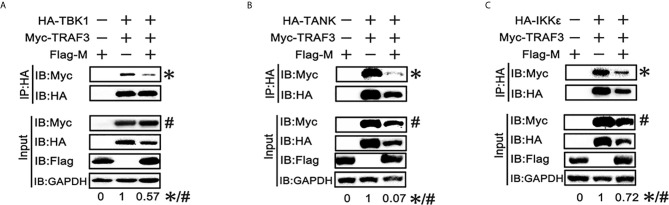
SRAS-CoV-2 M impedes the formation of TRAF3–TANK–TBK1 complex. **(A–C)** HEK293T cells were co-transfected with Myc-TRAF3, together with HA-tagged TBK1 **(A)**, TANK **(B)**, IKKϵ **(C)** with or without Flag-M for 24 h. The cell lysates were harvested and immunoprecipitated with anti-HA antibody. The cell lysates and the immunoprecipitants were analyzed by immunoblot. The relative band intensity (*/#) of co-immunoprecipitated TRAF3 in **(A–C)** was measured using ImageJ software.

SARS-CoV-2 M can interact with TRAF3, TBK1, IKKϵ and induce TBK1 degradation. Thus, we investigated whether it affects the formation of TRAF3–TANK–TBK1–IKKϵ complex, which is involved in IRF3 activation and IFN production (24). When the SARS-CoV-2 M protein was overexpressed, the binding of TRAF3 with TBK1, TANK and IKKϵ was reduced (**Figures 5A–C**), indicating that the SARS-CoV-2 M protein impairs the formation of TRAF3–TANK–TBK1–IKKϵ complex.

## Discussion

**Figure 6:**
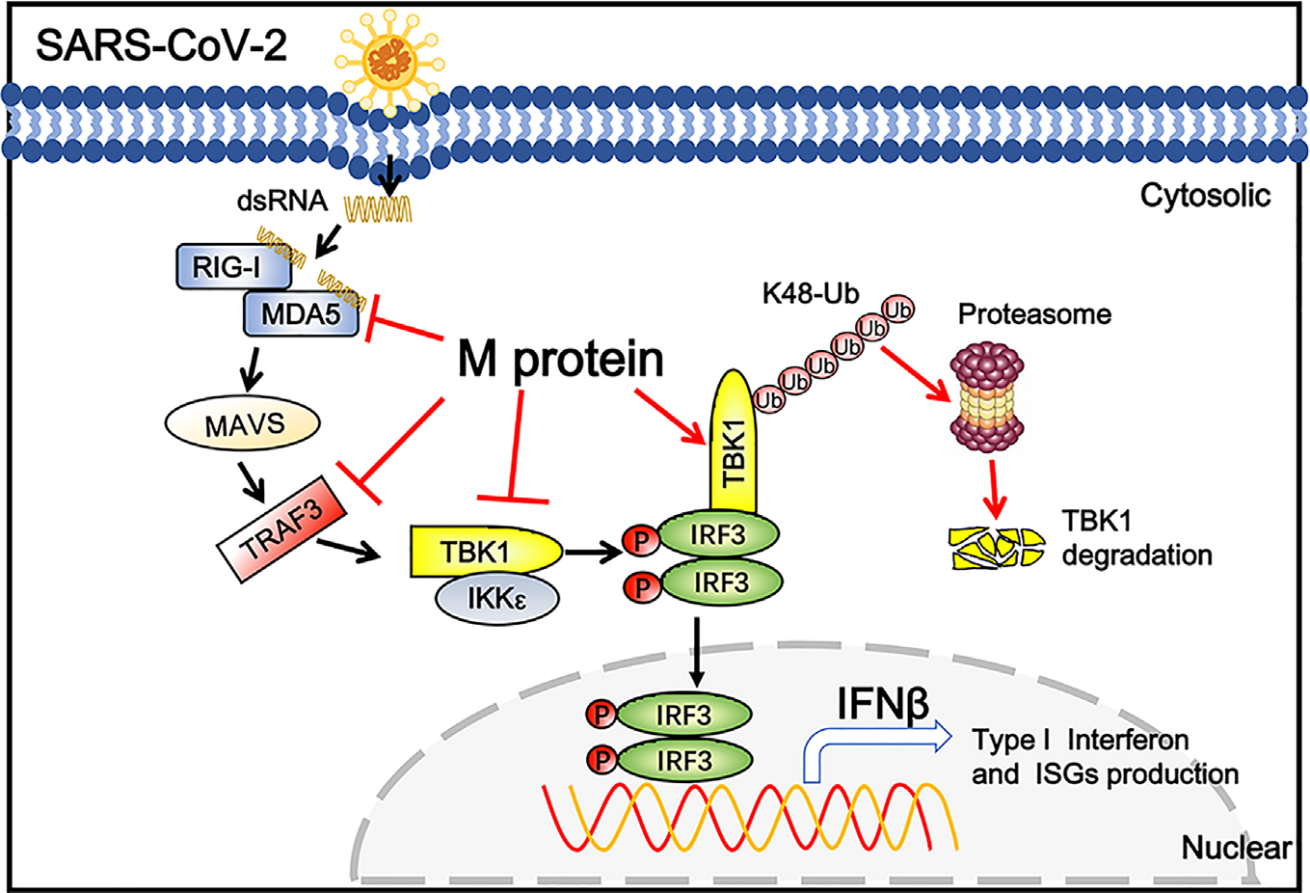
Schematic diagram of SARS-CoV-2 M inhibiting IFN-I signaling. M protein interacts with MDA5, TRAF3, TBK1, IKKϵ and degrade TBK1 *via* K48-linked ubiquitination, which suppresses the phosphorylation and nuclear translocation of IRF3 and blocks IFN signaling.

Coronaviral M protein, a glycosylated structural protein, is essential for the virion assembly (26, 27). SARS coronavirus M protein has been suggested to enhance the viral pathogen proliferation *via* inhibiting NF-κB and modulating apoptosis by interacting with phosphoinositide-dependent kinase-1 (PDK1) (28, 29). Previous studies have demonstrated that both SARS-CoV and MERS-CoV M can suppress the interferon production, in which SARS-CoV M protein impairs the formation of TRAF3–TANK–TBK1/IKKϵ complex (25), while MERS-CoV M interacts with TRAF3 and disrupts TRAF3-TBK1 association, resulting to suppressed IRF3 activation (30). Here, we identified the SARS-CoV-2 M protein as an inhibitor of the TBK1-mediated innate antiviral immune response. Our results demonstrated that SARS-CoV-2 M associates with TBK1 and degrades TBK1 *via* ubiquitin pathway, thereby inhibiting the phosphorylation of IRF3 and suppressing IFN-I production (**Figure 6**).

Recently, two studies have shown that SARS-CoV-2 M protein serves as an IFN antagonist, and overexpression of M protein significantly suppresses the IFN-I production triggered by SARS-CoV-2 and SeV (15, 16). However, whether M associates with TBK1 and inhibits TBK1-induced innate immunity is still controversial. One study concluded that SARS-CoV-2 M interacts with RIG-I, MDA5, MAVS and TBK1 to repress immune response (16). Another study reported that M inhibits the activation of the IFNβ promoter mediated by overexpression of RIG-I, MDA5, and MAVS, but not their downstream TBK1, and M can bind only MAVS but not RIG-I, MDA5 or TBK1 (15). Our results showed that SARS-CoV-2 M suppressed TBK1-induced IFNβ-Luc and ISRE-Luc reporters in a dose dependent manner, and the IRF3 phosphorylation induced by TBK1 was severely diminished in SARS-CoV-2 M treated cells, which provide consolidate evidence of the inhibitory effect of M on TBK1-induced innate immunity.

Zheng et al. (16) expressed both RIG-I and MDA5 abundantly in HEK293T cells, and the band of RIG-I precipitated by M was more weak than that of MDA5. The weak interaction of M and RIG-I may explain that only MDA5 was found to co-precipitate with SARS-CoV-2 M in our study. Further investigation is needed to confirm the interaction of SARS-CoV-2 M and RIG-I.

SARS-CoV M protein has been reported to bind with IKKϵ, impede IKKϵ induced IRF3 phosphorylation and TRAF3–IKKϵ interaction. Although SARS-CoV-2 M was found to be associated with IKKϵ and inhibited IKKϵ-induced IFNβ-Luc and ISRE-Luc reporters in our study, the phosphorylated IRF3 induced by IKKϵ was not affected by SARS-CoV-2 M co-expression. It is possible that SARS-CoV-2 M only affects the nuclear translocation, but not phosphorylation of IRF3 induced by IKKϵ, resulting to suppression of IFN production.

TBK1 is a key kinase of IFN-I signaling that is activated by the DNA and RNA sensors, such as RIG-I, MDA5, TLR3, and cGAS-STING (31–33); its activity must be strictly controlled to maintain appropriate IFN-I production (34–36). The ubiquitination of TBK1 is a key mechanism to modulate its activity. K63-linked ubiquitination of TBK1 is essential for its activation, while K48-linked ubiquitination mediates ubiquitin-dependent proteasomal degradation of TBK1 (22). In this study, we found that the SARS-CoV-2 M degraded TBK1 *via* K48-linked ubiquitination. During evolution, some viruses have acquired the capacity to take advantage of host factors to regulate the ubiquitin of RLR signaling molecules. For example, both SARS-CoV N and SARS-CoV-2 PLpro-TM proteins inhibit TRIM25-mediated K63-linked ubiquitination of RIG-I and suppress innate immune response (37, 38). Several deubiquitinating enzymes and E3 ubiquitin ligases have been shown to regulate the ubiquitin level of TBK1 and participate in innate immune response during virus infection (36, 39, 40). TRIM27 has been reported to interact and degrade TBK1 *via* K48-linked ubiquitination (39). However, SARS-CoV-2 M could not improve the recruitment of TRIM27 to TBK1 in our result (data not show). It is possible that SARS-CoV-2 M recruits other known or novel E3 ubiquitin ligases to induce TBK1 ubiquitination, which needs further investigation.

As SARS-CoV-2 M and SARS-CoV M protein shared approximately 91% amino acid identity, we also detected if SARS-CoV M could degrade TBK1. Results showed that co-transfection of SARS-CoV M suppressed TBK1 expression. However, MG132 did not affect the degradation of TBK1 mediated by SARS-CoV M protein, and co-expression of SARS-CoV M did not elevate the K48 ubiquitin level of TBK1 (**Figure S4**), indicating that SARS-CoV M did not degrade TBK1 *via* ubiquitination. Further investigation is needed to explore the mechanism of TBK1 degradation by the SARS-CoV M protein.

Taken together, the present study uncovered a novel mechanism by which SARS-CoV-2 M negatively regulates IFN-I signaling *via* interacting with MDA5, TRAF3, IKKϵ, and TBK1, and degrading TBK1 *via* K48-linked ubiquitination. Our findings provide deeper insight into the innate immunosuppression and pathogenicity of SARS-CoV-2.

## Data Availability Statement

The raw data supporting the conclusions of this article will be made available by the authors, without undue reservation.

## Author Contributions

QL and GT designed the research. LS, YZ, WW, PW, ZW, YY, and ZH performed the experiment. LS and WW analyzed data. LS prepared the manuscript. QL and GT revised the manuscript. All authors contributed to the article and approved the submitted version.

## Funding

This work was supported by the National Natural Science Foundation of China (81972873), the Pearl River Talent Plan in Guangdong Province of China (2019CX01N111), and the Science and Technology Innovation Project in Foshan, Guangdong Province, China (2020001000151), the 68th batch of first-class funding from China Postdoctoral Science Foundation (Grant No. 2020M680044 to GT).

## Conflict of Interest

The authors declare that the research was conducted in the absence of any commercial or financial relationships that could be construed as a potential conflict of interest.
