# Supplementary material for: SARS-CoV-2 Membrane Protein Inhibits Type I Interferon Production Through Ubiquitin-Mediated Degradation of TBK1
Source: Front Immunol. 2021 May 18;12:662989. doi: 10.3389/fimmu.2021.662989 (PMC8168463; doi:10.3389/fimmu.2021.662989)
Supplement: Supplementary file 1 [file Table_1.docx]

Supplementary Material

Supplementary Table 1. Primers used for quantitative real-time PCR (qPCR)

| **Genes** | **Sequences** | |
| --- | --- | --- |
|  | **Forward (5’ to 3’)** | **Reverse (5’ to 3’)** |
| *IFNα* | GCCTCGCCCTTTGCTTTACT | GGATCAGCTCATGGAGGACAGA |
| *IFNβ* | ATGACCAACAAGTGTCTCCTCC | GGAATCCAAGCAAGTTGTAGCTC |
| *ISG15* | TGGACAAATGCGACGAACC | CCCGCTCACTTGCTGCTT |
| *OAS1* | GGCAGAAATCACAGCAAG | GAGGGAGGGAGTTCACAT |
| *SOCS1* | ACGCACTTCCGCACATTCC | TCCCGAGGCCATCTTCACG |
| *GAPDH* | GAGTCAACGGATTTGGTCGT | TGGGATTTCCATTGATGACA |
| *TBK1* | CGGAGACCCGGCTGGTATAA | ATCCACTGGACGAAGGAAGC |


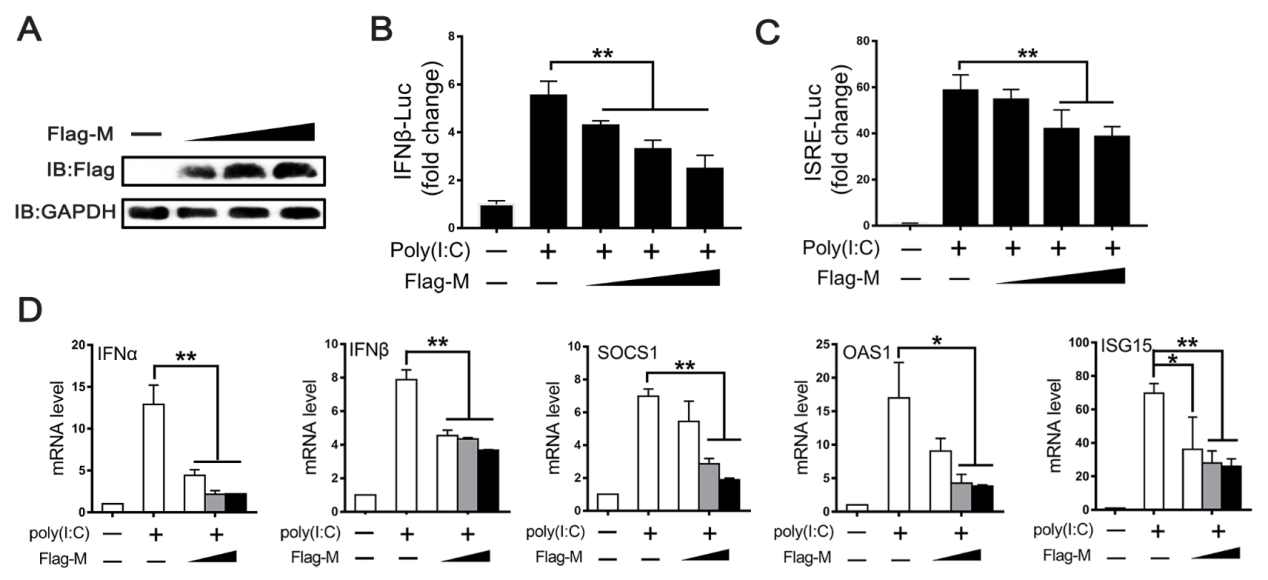


**Figure S1. SARS-CoV-2 M inhibits IFN-I signaling.**

**(A)** Flag tagged empty vector or increasing amount of Flag-M (0.2, 0.4 and 0.8 μg ) expression plasmids were transiently transfected into HEK293T cells and analyzed by immunoblot using anti Flag antibody. GAPDH was used as an internal loading control.

**(B, C)** Flag tagged empty vector or increasing amount of Flag-M (0.2, 0.4 and 0.8 μg ) expression plasmid was co-transfected with an IFNβ or ISRE reporter plasmid, along with 1 μg/mL poly(I:C). After 24 h, cells were harvested for dual-luciferase IFNβ or ISRE reporter assay. Bars represent the mean of three biological replicates (n=3) and all data were expressed as mean ± SE,

**(D)** Flag tagged empty vector or increasing amount of Flag-M (0.2, 0.4 and 0.8 μg ) expression plasmid was co-transfected with 1 μg/mL poly(I:C). After 24 h, cells were harvested and relative mRNA levels of type I IFN and ISGs genes expression were analyzed by qPCR. GAPDH was used as a normalizer.

**
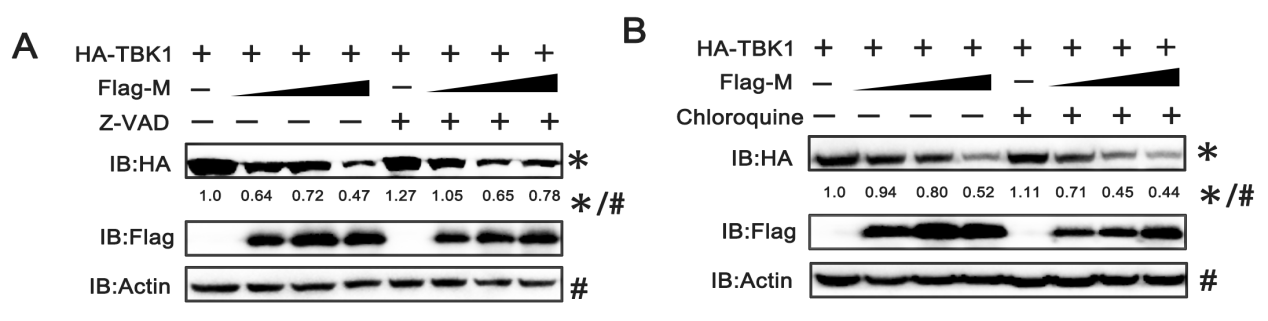
**

**Figure S2. TBK1 degradation mediated by M protein was not effected by caspase and lysosome inhibitors**

**(A, B)** HEK293T cells were transfected with a control plasmid or increasing doses of Flag-M (0.25, 0.5, 1 μg) and HA-TBK1 plasmids. After 24 h, cells were treated with 10 μM Z-VAD **(A)** or chloroquine **(B)** for 6 h. The cell lysates were analyzed by immunoblot.


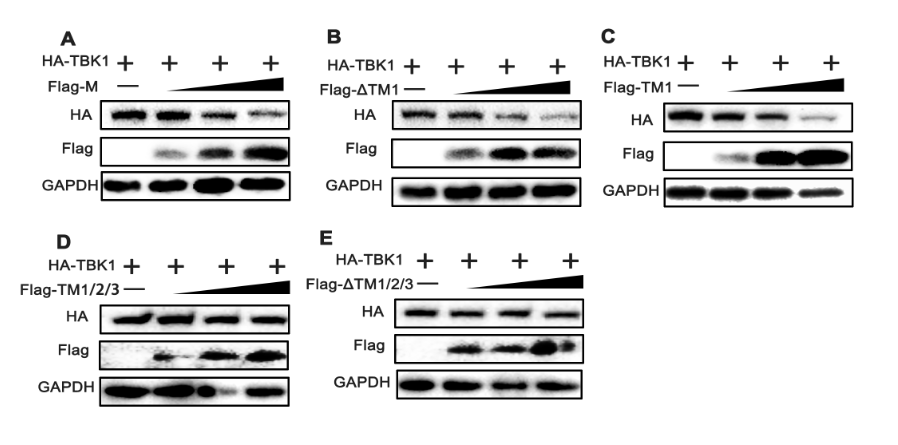


**Figure S3. ΔTM1 and TM1 truncations of SARS-CoV-2 M induces TBK1 degradation**

**(A-E)** Increasing doses (0.25, 0.5 and 1 μg) of Flag-M and its truncations expression plasmids were co-transfected with 0.5 μg TBK1. After 24 h, cells were harvested for immunoblot analysis.


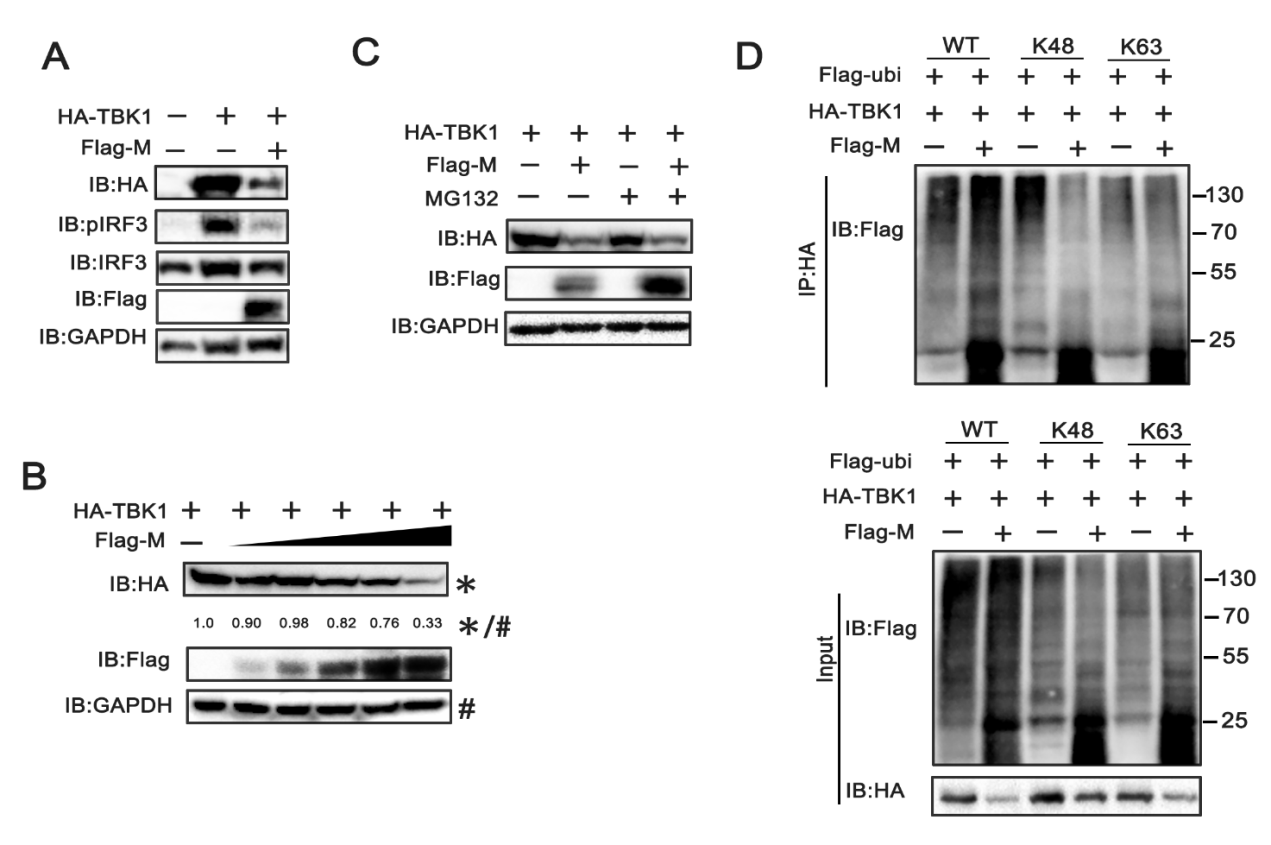


**Figure S4. SARS-CoV-M degraded TBK1 not via ubiquitination pathway**

**(A)** HEK293T cells were transfected with SARS-CoV M together with HA-tagged TBK1, which was applied to activate IRF3. Proteins were extracted 24 h after transfection. The indicated proteins were analyzed by immunoblot. GAPDH was detected as a loading control.

**(B)** HEK293T cells in 6-well plate were transfected with a control plasmid or increasing doses of plasmid expressing SARS-CoV M (0.25, 0.5, 1, 2 and 4 μg), along with HA-TBK1 plasmids for 24 h. The cell lysates were analyzed by immunoblot. GAPDH was detected as a loading control.

**(C)** HEK293T cells were transfected with a control plasmid or increasing doses of SARS-CoV M and HA-TBK1 plasmids. After 24 h, cells were treated with 20 μM MG132 or DMSO for 6 h. The cell lysates were analyzed by immunoblot.

**(D)** Co-immunoprecipitation and immunoblot analysis of extracts from HEK293T cells transfected with TBK1 with or without SARS-CoV M as well as Flag-Ub (WT, K48-linked, or K63-linked) expression plasmids as indicated.
